# A Retrospective Evaluation of Critical Care Blood Culture Yield – Do Support Services Contribute to the “Weekend Effect”?

**DOI:** 10.1371/journal.pone.0141361

**Published:** 2015-10-22

**Authors:** Ben Morton, Shankara Nagaraja, Andrea Collins, Shaun H. Pennington, John D. Blakey

**Affiliations:** 1 Department of Clinical Sciences, Respiratory Infection Group, Liverpool School of Tropical Medicine, Liverpoool, United Kingdom; 2 Aintree University Hospitals NHS Foundation Trust, Liverpool, United Kingdom; 3 Royal Liverpool University Hospital, Liverpool, United Kingdom; Azienda Ospedaliero-Universitaria Careggi, ITALY

## Abstract

**Background:**

The “weekend effect” describes an increase in adverse outcomes for patients admitted at the weekend. Critical care units have moved to higher intensity working patterns to address this with some improved outcomes. However, support services have persisted with traditional working patterns. Blood cultures are an essential diagnostic tool for patients with sepsis but yield is dependent on sampling technique and processing. We therefore used blood culture yield as a surrogate for the quality of support service provision.

We hypothesized that blood culture yields would be lower over the weekend as a consequence of reduced support services.

**Methods:**

We performed a retrospective observational study examining 1575 blood culture samples in a university hospital critical care unit over a one-year period.

**Results:**

Patients with positive cultures had, on average, higher APACHE II scores (p = 0.015), longer durations of stay (p = 0.03), required more renal replacement therapy (p<0.001) and had higher mortality (p = 0.024). Blood culture yield decreased with repeated sampling with an increased proportion of contaminants. Blood cultures were 26.7% less likely to be positive if taken at the weekend (p = 0.0402). This effect size is the equivalent to the impact of sampling before and after antibiotic administration.

**Conclusions:**

Our study demonstrates that blood culture yield is lower at the weekend. This is likely caused by delays or errors in incubation and processing, reflecting the reduced provision of support services at the weekend. Reorganization of services to address the “weekend effect” should acknowledge the interdependent nature of healthcare service delivery.

## Introduction

The “weekend effect” describes impaired outcomes for hospital patients over the weekend compared with weekdays. These adverse outcomes include greater mortality associated with emergency admissions [1], elective admissions [2] and after elective surgical procedures [3]. Patients admitted at the weekend also have a longer length of stay [4] and a lower likelihood of receiving urgent specialist intervention [5]. Most hospitals have fewer, and more junior staff working at the weekend [6]. Many critical care units have changed to a higher-intensity staffing model; with some improvement in outcome for patients admitted at the weekend [7,8]. However, differences in outcome persist; this suggests other links in the chain providing care to the critically ill may remain weak, including support services such as laboratory provision and radiology [9].

Blood cultures are an essential diagnostic tool for all patients with sepsis, recommended by international guidelines [10]. Blood cultures are the current standard in detection of blood stream infection [11]. However, most blood cultures (~93% in general hospital population) are negative for bacterial growth with high contamination rates (false positives) in cultures with growth [12,13]. Thus, antibiotic therapy decisions are frequently guided by clinical status rather than diagnostic results [14,15] Prediction tools to guide clinicians on appropriate sampling do improve diagnostic yield [16,17] but test sensitivity and specificity remains heavily dependent on sampling technique, handling and processing [18]. As blood culture yield is affected by delays in handling and processing of samples [19], we chose to use it as a marker for variation in the quality of hospital support services.

We set out to compare the yield of blood cultures taken at the weekend compared with weekdays. Our aim was to establish if the reduced level of service at the weekend was associated with a change in blood culture yield. If present, the weekend effect could have implications for resourcing of support services, especially with the introduction of newer and more complex investigations such as the use of molecular based techniques in microbiology[20].

## Materials and Methods

### Study design

We conducted an observational retrospective, single-centre cohort study at University Hospital Aintree a 720-bedded tertiary-referral centre in Liverpool, United Kingdom. This study was undertaken as part of a service evaluation and approved internally through the audit department processes at Aintree University Hospital NHS Foundation Trust. No consent was taken for this study; data were retrospective and analysed anonymously. The cohort was composed of patients admitted to a critical (level 2 and 3) care unit (19 beds) between 1^st^ April 2011 and 31^st^ March 2012. The unit accepts medical and surgical admissions, and the hospital is a tertiary referral centre for upper gastrointestinal, hepatobiliary, trauma, maxillofacial and otolaryngeal surgical patients. The department’s blood culture policy adheres to Centre for Disease Control sampling recommendations [21]. Samples, once taken, are transported directly to a central laboratory by health care staff, in keeping with other UK teaching hospitals. If a delay in transportation is anticipated, the hospital porter is called as contingency. Our hospital uses the BD BACTEC™ instrumented blood culture system.

Patients who had one or more blood culture samples taken on critical care during the study period were included. We defined a positive blood culture result as contaminated in the event of unpaired isolation of *Staphylococcus capitis*, *Staphylococcus epidermidis*, *coagulase negative staphylococcus*, *Propionibacterium* species, *Diphtheroid bacilli*, Gram positive bacilli or non-haemolytic *Streptococcus* species in line with previous work [22].

The primary analysis was blood culture yield on weekdays versus weekends. Secondary analyses investigated blood culture yield with respect to factors previously seen to be influential, such as repeated sampling and source of infection, to provide context for any effect seen for the primary analysis.

### Data collection

Blood culture data was retrieved from the electronic laboratory database; only samples sent from the critical care area were included. Our unit contributes to the case-mix programme for the England, Wales and Northern Ireland, coordinated by the Intensive Care National Audit and Research Centre (ICNARC). We used this data to populate the demographic characteristics of our cohort.

### Statistical analysis

Data is presented as median and interquartile range (IQR) for continuous variables and as absolute or relative frequencies for categorical variables. Data analysis used Pearson Chi-square and Mann-Whitney tests undertaken using SPSS version 21 (IBM, Illinois).

## Results

### Population under study

A total of 1368 patients were admitted to the critical care unit during the study period. Of these patients, 517 had a blood culture sample taken (1575 samples in total). Paired samples (defined as two samples received by laboratory within 24hrs) were taken on 1070 occasions (67.9%). One hundred and thirty three (25.7%) patients had at least one positive blood culture. Of 1575 samples taken, a total of 245 were positive (15.6%) with multiple samples taken in some patients. Demographic information is detailed in Table 1. Of the 517 patients, 148 were admitted at the weekend compared to 369 on a weekday. There was no difference between mortality length of stay (median 4 vs. 4, p = 0.194) or (32.8% vs. 32.4%, p = 0.414) in weekend compared to weekday admissions in this cohort of patients who met the criteria for blood culture sampling. We did not find that the proportion of negative to positive to contaminated blood cultures differed according to the number of admissions per day (S1 Fig).

**Table 1:** Patient demographics and outcomes according to blood culture positivity.

	Patients with		P value
	≥1 Positive culture (133)	Negative culture (384)	
Surgical admissions (n)	38	89	
Medical admissions (n)	96	294	
Age (median, IQR)	63 (51–71)	63 (50–73)	0.83
Gender (%male)	62.7% (183)	52.3% (201)	0.031
Advanced respiratory support (n,%)	71 (53.3%)	186 (48.4%)	0.33
Length of stay (median, IQR)	5(7)	4 (6)	0.030
APACHE II (median, IQR)	18 (9)	17 (8)	0.015
Renal replacement therapy (n, %)	33 (24.8%)	44 (11.5%)	0.00019
Died (n,%)	54 (40.6%)	115 (29.9%)	0.024

Table describes demographic and outcome data for patients split by blood culture result: one or more positive blood cultures vs. all cultures negative. Data is presented as frequency (n), median, interquartile range (IQR) and percentage (%). Advanced respiratory support is the number of days requiring ventilation, APACHE II is the Acute Physiology and Chronic Health Evaluation II score—used at admission for patients in our critical unit.

### Blood culture yield and organism grown

**Fig 1:**
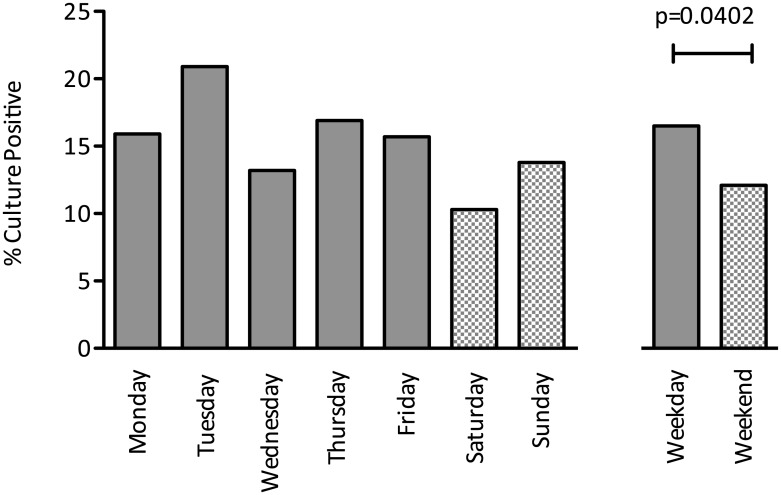
Blood culture positives (%) according to day of sample. The percentage of positive blood cultures is shown for each day with a grouping for weekday and weekend. Mann-Whitney U test demonstrates a significant difference (p = 0.0402) between weekday (16.5% positive) and weekend (12.1% positive) samples. This equates to a 26.7% decrease in positive cultures at the weekend.

**Fig 2:**
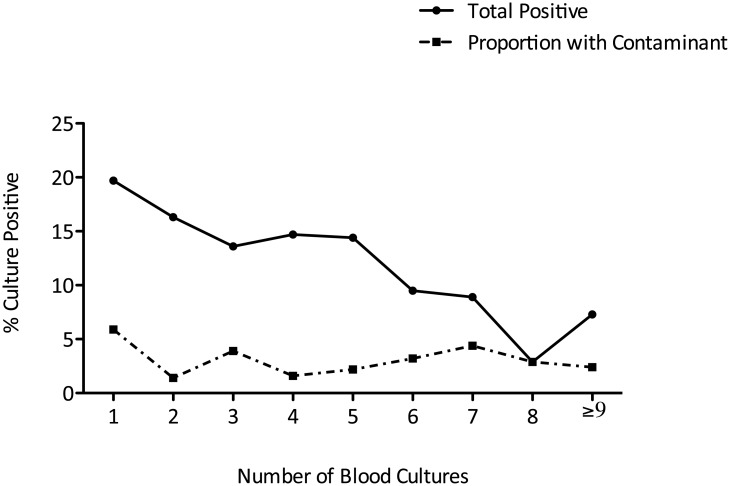
Blood culture positives and contaminants (false positives) (%) in sequential samples. The change in the percentage of positive blood cultures (solid black line) and the percentage positive cultures considered a contaminant (broken black line) is shown at each sample point.

**Fig 3:**
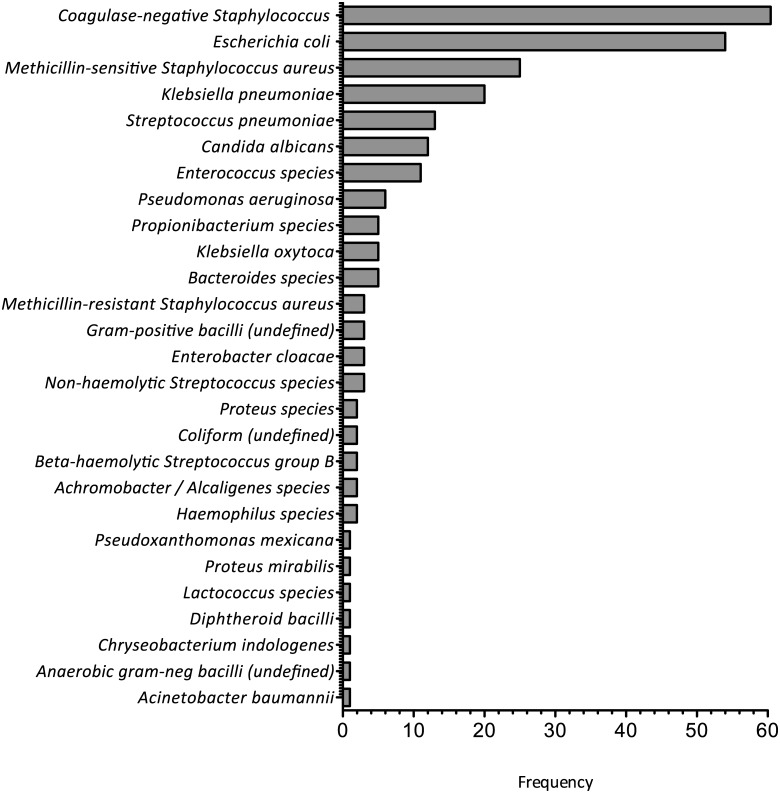
Type and frequency of organisms grown in patient cohort. The growth and frequency of organisms defined according to laboratory analysis is shown in descending order. Total number of positive cultures is 245. NB: there were 10-paired samples of coagulase-negative *Staphylococcus (CNS)*. A paired sample growing *CNS* is usually pathogenic [22] and not considered to be a contaminant.

Blood cultures were 26.7% more likely to be positive if received by the laboratory on a weekday compared with the weekend (absolute yields: 16.5% vs. 12.1%, p = 0.0402) (Fig 1). This observation was consistent with samples taken on Bank Holidays during the study period (1 positive from 34 samples, 2.9%). The number of positive cultures declined with repeated sampling after the first sample whilst the number of suspected contaminants remained broadly similar (Fig 2). The most common isolate was coagulase-negative *Staphylococcus* (Fig 3). There was no significant difference in blood culture contamination between samples taken at the weekend compared to weekdays (4.6% vs. 3.3%, p = 0.234).

### Source of Infection

**Fig 4:**
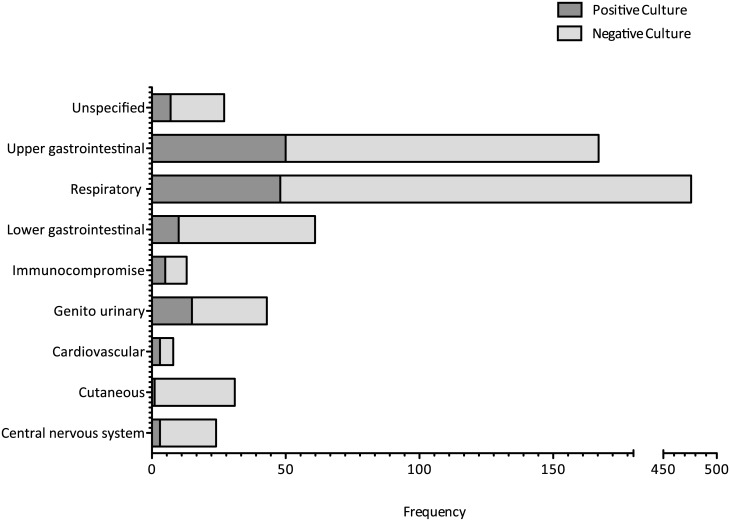
Blood culture results by source of clinical infection (%). The number of cultures with and without growth according to the source of clinical infection. Source of infection is split into systems for convenience. NB the scale is split for respiratory infection—the most common indication for blood culture in our cohort.

Respiratory tract infection was the most common indication for culture, with 10.1% of cultures positive (48/476). Samples with higher yield results were found in genito-urinary infection 15/43 (34.9%), upper gastrointestinal infection 50/167 (29.9%) and immunocompromised patients with 5/13 (38.5%) (Fig 4).

## Discussion

Blood cultures are instructive when positive but often reveal no growth or are contaminated. It is known that culture samples, if not processed in time, lead to suboptimal results [19]. We postulated that blood culture yield would be lower at the weekend, possibly reflecting the reduced resources allocated to support services at this time, and highlighting the range of potential contributors to the “weekend effect”.

We found a significantly lower yield in blood cultures taken at the weekend when compared to those taken on weekdays. This finding is in keeping with other studies examining weekend access to investigations and procedures [9,23]. Surviving Sepsis Guidelines recommend two sets of blood cultures with the diagnosis of sepsis followed by early antibiotic administration [10]. It is well documented that sensitivity of blood cultures is diminished after antibiotics are given [24]. To put the magnitude of the weekend effect for blood cultures into context; there was a 4.4% absolute reduction in the proportion of cultures that were positive between weekdays and the weekend compared to a 3% drop between the second and third cultures (third culture typically taken after antibiotic administration).

Support services such as microbiology have tended to maintain traditional working patterns, with a limited weekend on-call service. Our study suggests that this practice could lead to less accurate diagnosis in sepsis; lack of evidence based antibiotic prescribing may have a negative impact on patient outcome. Critical care units have increased the numbers of both junior and senior staff at the weekend with a positive impact on patient outcome [7,8]. Is it now time for supporting services to follow suit?

Our study demonstrates a diminishing yield with repeated blood culture sampling. Early administration of antibiotics in suspected infection is vital to reduce mortality [25] but the sensitivity of subsequent blood culture sampling is reduced [24].

Respiratory infection is a major cause of admission to critical care (44% of sepsis) [26] and was the most common indication for blood culture in our cohort. This finding is in keeping with other studies demonstrating variation in diagnostic yield by site of sepsis [14,15]. Focus on alternative respiratory samples (sputum, tracheal aspirate or bronchoalveolar lavage) may improve microbiological yield [27]; however, prior antibiotic administration decreases sensitivity.

Our suspected contamination rate of 3.6% (22.9% of positive cultures) is in line with other reports [22]; sample contamination can lead to inappropriate antibiotic therapy, increased length of stay and cost [28]. Fig 2 demonstrates similar contamination rates irrespective of sample point; as the overall positive rate falls, contaminants comprise a greater proportion of yield—the clinician should be mindful of this when ordering repeated tests.

In our sample set, patients with positive cultures were sicker at admission (higher APACHE II scores), required longer lengths of stay in critical care and were more likely to require renal replacement therapy. In our unselected cohort we found that bacteraemia was significantly associated with mortality (Table 1).

This novel study attempts to unpick reasons for the “weekend effect” and highlights the need for improved support services for acutely unwell patients. We present a data set of 1575 cultures in a general critical care unit over a one-year period. Limitations of this study are that it is based in a single site, paired samples were not universal and the culture transfer times and conditions (bed to laboratory to incubator) were not available. It is possible that delay to incubation of samples at the weekend could have impacted upon results. However, critical care staffing levels are maintained at the weekend so it is unlikely that delay in transportation to the laboratory would occur and we did not find a significant increase in blood culture contamination in weekend samples. This study was not designed to assess the effect of blood culture results on antibiotic prescribing; hence the laboratory and clinical data were not correlated. In addition, we were unable to measure the inter-relationship between antibiotic administration and blood culture sampling in this study so could not determine if a systematic difference in prior antibiotic administration lead to a higher rate of negative cultures at the weekend. However, it is plausible that weekend working is more likely to lead to delays in antibiotic administration and thus higher positivity rates might be expected.

Our study raises a number of questions related to current standard practice in this area. Decreased blood culture positivity is unlikely to be due to a single adverse factor. Thus, there is a need for further research to investigate what aspects of laboratory provision, portering and other support services would increase yield from specific investigations. Additional research could also seek to establish whether any improvements in yield from such microbiological investigations would translate into improved clinical outcomes.

Our study adds to the increasing body of evidence of impaired patient outcome due to the “weekend effect”. Blood cultures positivity rate may act as a surrogate indicator of the broader issue of decreased access to, or quality of, support services at weekends. We encourage healthcare policy makers and managers to consider the implications of weekend working patterns both in terms of direct patient care and supporting services.

## Supporting Information

S1 Fig Primary blood culture results by number of admissions per day. Figure displays the proportion of primary blood culture results that are positive, negative or contaminated according to the number of admissions per day. Horizontal axis displays number of admission per day and the number (N) of days on which this number of admissions occurred. The vertical axis displays the total number of blood culture samples.
(TIFF)
