# Supplementary figures and images for: A Retrospective Evaluation of Critical Care Blood Culture Yield – Do Support Services Contribute to the “Weekend Effect”?
Source: PLoS One. 2015 Oct 22;10(10):e0141361. doi: 10.1371/journal.pone.0141361 (PMC4619625; doi:10.1371/journal.pone.0141361)

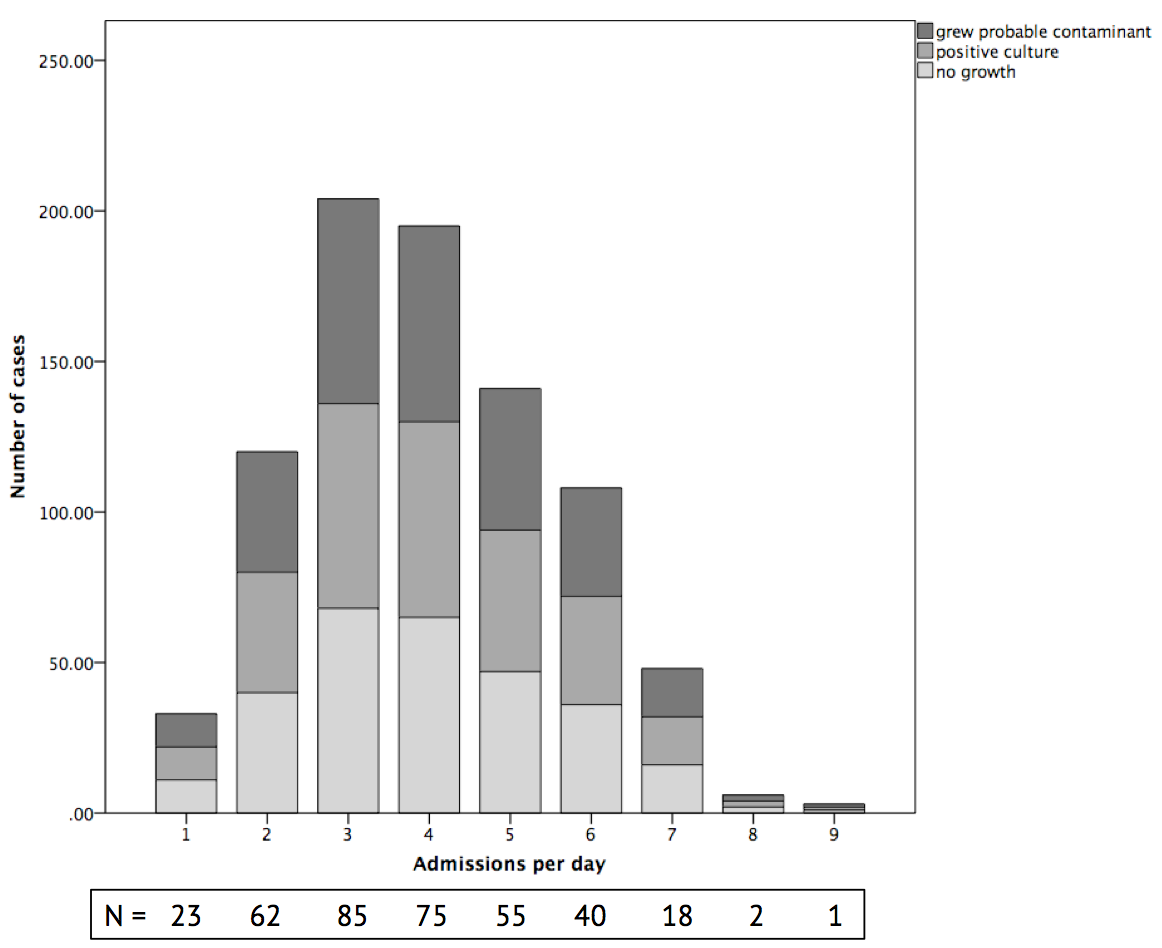

Supplement: S1 Fig — Figure displays the proportion of primary blood culture results that are positive, negative or contaminated according to the number of admissions per day. Horizontal axis displays number of admission per day and the number (N) of days on which this number of admissions occurred. The vertical axis displays the total number of blood culture samples. (TIFF) [file pone.0141361.s001.tiff]
